# Alzheimer’s cerebrospinal biomarkers from Lumipulse fully automated immunoassay: concordance with amyloid-beta PET and manual immunoassay in Koreans: CSF AD biomarkers measured by Lumipulse in Koreans

**DOI:** 10.1186/s13195-020-00767-3

**Published:** 2021-01-12

**Authors:** Sohee Moon, Sujin Kim, Sakulrat Mankhong, Seong Hye Choi, Manu Vandijck, Vesna Kostanjevecki, Jee Hyang Jeong, Soo Jin Yoon, Kyung Won Park, Eun-Joo Kim, Bora Yoon, Hee Jin Kim, Jae-Won Jang, Jin Yong Hong, Dong-Ho Park, Leslie M. Shaw, Ju-Hee Kang

**Affiliations:** 1 Department of Pharmacology and Hypoxia-related Disease Research Center, College of Medicine, Inha University, Room 1015, 60th Anniversary Hall, 100, Inha-ro, Nam-gu, Incheon, 22212 South Korea; 2 Department of Kinesiology, Inha University, Incheon, 22212 South Korea; 3 Program in Biomedical Science and Engineering, Inha University, Incheon, 22212 South Korea; 4 Department of Neurology, College of Medicine, Inha University, Incheon, 22332 South Korea; 5 Fujirebio-Europe N.V., Technologiepark 6, 9052 Ghent, Belgium; 6 Department of Neurology, Ewha Womans University Mokdong Hospital, Ewha Womans University School of Medicine, Seoul, 07985 South Korea; 7 Department of Neurology, Eulji University Hospital, Eulji University School of Medicine, Daejeon, 35233 South Korea; 8 Department of Neurology, Dong-A Medical Center, Dong-A University College of Medicine, Busan, 49201 South Korea; 9 Department of Neurology, Pusan National University Hospital, Pusan National University School of Medicine and Medical Research Institute, Busan, 49241 South Korea; 10 Department of Neurology, Konyang University College of Medicine, Daejeon, 35365 South Korea; 11 Department of Neurology, Samsung Medical Center, Sungkyunkwan University School of Medicine, Seoul, 06351 South Korea; 12 Department of Neurology, Kangwon National University Hospital, Chuncheon, 24289 South Korea; 13 Department of Neurology, Yonsei University Wonju College of Medicine, Wonju, 26426 South Korea; 14 Department of Pathology and Laboratory Medicine, Perelman School of Medicine, University of Pennsylvania, Philadelphia, 19104 PA USA

**Keywords:** Alzheimer’s disease (AD), Cerebrospinal fluid (CSF), Lumipulse fully automated immunoassay, β-Amyloid positron emission tomography (Aβ-PET), Biomarker, Korean

## Abstract

**Background:**

Alzheimer’s disease (AD) cerebrospinal fluid (CSF) biomarker cutoffs from immunoassays with low interlaboratory variability in diverse ethnic groups are necessary for their use in clinics and clinical trials. With lack of cutoffs from fully automated immunoassay platforms in diverse races, the aim of this study is to evaluate the clinical utility of CSF AD biomarkers from the Lumipulse fully automated immunoassay based on β-amyloid (Aβ) positron emission tomography (PET) status comparing with these from two manual immunoassays, in Koreans.

**Methods:**

Among 331 Korean participants enrolled from a prospective, 3-year longitudinal observational study of the validation cohort of Korean Brain Aging Study for the Early Diagnosis and Prediction of AD, 139 (29 CN, 58 SCD, 29 MCI, and 23 AD) provided CSF and 271 underwent baseline amyloid PET (*n* = 128 with overlapping CSF and Aβ-PET, and 143 without CSFs). Three annual cognitive and neuropsychiatric function tests were conducted. Aβ42, Aβ40, total-tau, and phosphorylated-tau_181_ were measured by Lumipulse fully automated immunoassay and two manual immunoassays (INNO-BIA AlzBio3, INNOTEST). Clinical utility of CSF biomarker cutoffs, based on 128 participants with Aβ-PET, was evaluated.

**Results:**

Cognitive and neuropsychological scores differed significantly among the groups, with descending performance among CN>SCD>MCI>AD. Biomarker levels among immunoassays were strongly intercorrelated. We determined the Aβ-PET status in a subgroup without CSF (*n* = 143), and then when we applied CSF biomarker cutoffs determined based on the Aβ-PET status, the CSF biomarkers (cutoffs of 642.1 pg/mL for Aβ42, 0.060 for Aβ42/Aβ40, 0.315 for t-tau/Aβ42, and 0.051 for p-tau/Aβ42, respectively) showed good agreement with Aβ-PET (overall AUC ranges of 0.840–0.898). Use of the Aβ-PET-based CSF cutoffs showed excellent diagnostic discrimination between AD and CN (Aβ42, Aβ42/Aβ40, t-tau/Aβ42, and p-tau/Aβ42) with overall AUC ranges of 0.876–0.952. During follow-up, participants with AD-like CSF signature determined by Aβ-PET-based cutoffs from Lumipulse showed rapid progression of cognitive decline in 139 subjects, after adjustment for potential confounders, compared with those with a normal CSF signature.

**Conclusion:**

CSF AD biomarkers measured by different immunoassay platforms show strong intercorrelated agreement with Aβ-PET in Koreans. The Korean-specific Aβ-PET-based CSF biomarker cutoffs measured by the Lumipulse assay strongly predicts progression of cognitive decline. The clinical utility of CSF biomarkers from fully-automated immunoassay platforms should be evaluated in larger, more diverse cohorts.

## Background

Given the pathologic characterization of Alzheimer’s disease (AD) by amyloid-β (Aβ) plaques and neurofibrillary tangles, measurement of AD biomarkers amyloid beta (1–42 and 1–40) (Aβ42, Aβ40), total tau (t-tau), and phosphorylated tau at Thr181 (p-tau) in cerebrospinal fluid (CSF) is recommended for accurate AD diagnosis and research [1, 2]. These biomarkers have been widely appreciated that the AD-like feature of “core” CSF AD biomarkers characterized by a lower Aβ42 and higher t-tau or p-tau levels in the CSF of patients with AD, compared with that of healthy older adults, reflects the abnormal Aβ plaque burden and tau pathology. Although the concentrations of each biomarker measured by single-plex or multiplex immunoassay platforms are not interchangeable, their concentrations are highly correlated and diagnostic performance is comparable [3–6]. In a qualified laboratory in which CSF AD biomarkers are measured routinely, the intra-laboratory precision for single-plex enzyme-linked immunosorbent assay (ELISA) method or multiplex xMAP-Luminex is excellent [7, 8]. Nevertheless, manual immunoassay-based concentration of Aβ42 and tau proteins across laboratories varies, even using equivalent CSF samples with standardization of preanalytical variables [8–10] or with unified test procedure following comprehensive standard operating procedures (SOPs) [11]. Implementation of unified SOPs in an experienced laboratory may decrease variability in determining their internal cutoffs for AD diagnosis; however, manual assays have inherent sources of analytical variability. Therefore, a fully automated immunoassay for routine clinical practice for CSF biomarker-based diagnosis of AD is desirable. Furthermore, using the reference method procedure such as liquid chromatography tandem mass spectrometry and certified reference material (CRM) for Aβ42 using neat CSF was recently introduced to harmonize immunoassays across platforms, to eliminate systemic bias in CSF Aβ42 levels, and across kit lots [12]. Currently, fully automated immunoassay systems have been developed [13], including the Elecsys developed by Roche Diagnostics (Rotkreuz, Switzerland) and the Lumipulse developed by Fujirebio (Fujirebio Europe, Gent, Belgium), which show high concordance with amyloid positron emission tomography (PET) classification [14–16].

With lack of cutoffs from fully automated immunoassay platforms in diverse races, the possibility of the universal cut-offs in CSF AD biomarkers should be evaluated. Herein, we analyzed CSF samples from a validation cohort in the Korean Brain Aging Study for the Early Diagnosis and Prediction of Alzheimer’s Disease (KBASE-V study) using a fully automated immunoassay Lumipulse G and two manual immunoassay platforms: xMAP-Luminex INNOBIA-AlzBio3 multiplex assay (Luminex) and ELISA with INNOTEST kit (INNOTEST) for Aβ42, Aβ40 (only for INNOTEST and Lumipulse G), t-tau, or p-tau. We evaluated, in Koreans for the first time, the overall agreement of these core CSF AD biomarkers with amyloid PET results, the correlations among the CSF biomarker levels measured with these three platforms, and the diagnostic performance of each biomarker using a cutoff based on Aβ-PET status. We also assessed the predictability of baseline CSF biomarkers for cognitive decline over 3 years.

## Methods

### Participants

Among 331 participants from nine memory clinics across South Korea (KBASE-V study participants; 71 cognitively normal (CN), 99 subjective cognitive decline (SCD), 89 mild cognitive impairment (MCI), and 72 probable AD), 139 (29 CN, 58 SCD, 29 MCI, and 23 AD) agreed to provide CSF. Supplementary information (Supplementary Method 1**)** presents the criteria for clinical diagnosis of SCD, MCI, and AD, and exclusion criteria [17, 18]. Clinical assessments conducted at baseline and every year for 3 years included the Korean version of the Mini-Mental State Examination (MMSE) in the CERAD assessment packet [17]; the Subjective Memory Complaints Questionnaire [19]; the Geriatric Depression Scale (GDS); the CDR and Global Deterioration Scale [18]; the Blessed Dementia Scale-Activities of Daily Living (BDS-ADL); and comprehensive neuropsychological testing. Demographic information including age, gender, and education years was collected for all participants. Peripheral blood was drawn for ApoE genotyping and laboratory tests. Ethical approval was given by the Institutional Review Board of each center (INHAUH 2015-03-021). All participants or their legal representatives voluntarily agreed to participate and provided written informed consent. The datasets used and/or analyzed during the current study are available from the corresponding author on reasonable request.

### CSF collection and AD biomarker analysis using manual immunoassay systems

A total of 139 participants underwent lumbar puncture in the morning. CSF was drawn in 15 mL polypropylene (PP) tubes (Falcon, Corning Science, NY, USA) and immediately centrifuged at 2000×*g* for 10 min at room temperature (RT). The supernatant (~ 10 mL) was frozen on dry ice and transferred to the laboratory at Inha University for AD biomarker analyses. Transported CSF was thawed at RT, gently mixed with a PP tip pipette, 0.4 mL aliquots divided into 0.5-mL PP tubes (Sarstedt AG & Co., Nümbrecht, Germany), and stored at − 80 °C until analysis. We applied two manual immunoassay platforms (INNOTEST and Luminex-AlzBio3) and one fully automated immunoassay to measure AD biomarker levels, as previously described (Additional file: Supplementary Method 2) [20].

### Fully automated immunoassays for CSF AD biomarkers using Lumipulse

Using Lumipulse G1200 fully automated immunoassay system with Lumipulse® G p-tau_181_, t-tau, Aβ42, or Aβ40 kit (Fujirebio Europe), additional CSF aliquots were analyzed. The Lumipulse G instruments use single analyte, ready-to-use, immunoreaction cartridges with a throughput of 60 and 120 tests/h for the G600II and the G1200 instruments, respectively. The analyte is captured specifically by antibody-coated microparticles before the biotinylated detection antibodies (streptavidin labeled with alkaline-phosphatase, i.e., for Aβ42 and t-tau assays) or ALP labeled detection antibodies (i.e., for Aβ40 and p-tau assays) and substrate are added, each after a thorough washing step. Established monoclonal antibodies were used in the set-up for the Lumipulse G assays. Analysis of the CSF samples (from storage vials) was accomplished with a Lumipulse G 1200 series instrument using the Lumipulse G Aβ42 (CRM standardized), Aβ40, t-tau, and p-tau assays at the Fujirebio Gent facility. The concentrations were within the assay’s measurement range, except for 16 samples (all Aβ-PET negative) in which the t-tau measurements were all below the limit of quantitation (141 pg/mL). The excellent analytical performance for the assays has been described previously [21–23].

### Amyloid positron emission tomography

**Fig. 1:**
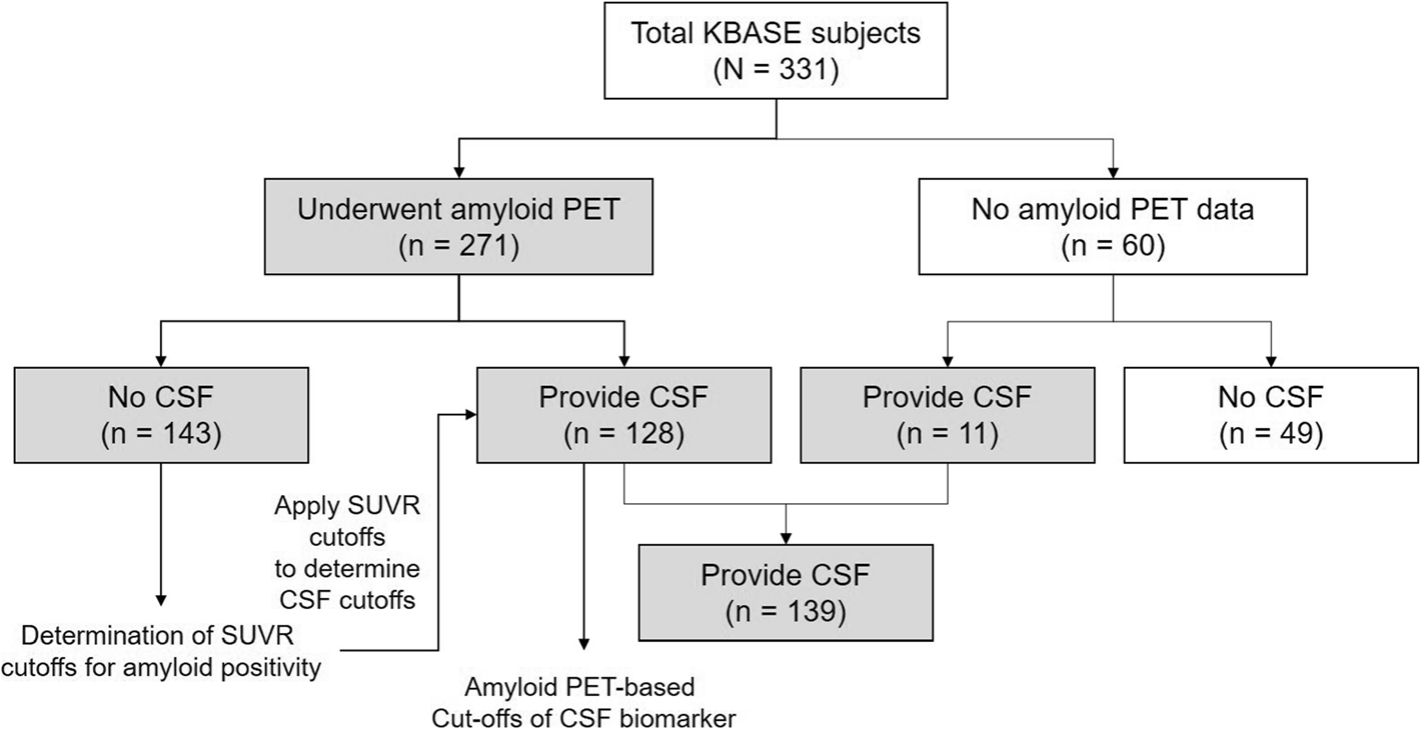
Summarized flow chart of study population. Among 271 subjects with amyloid PET imaging, in the participants with amyloid PET imaging but without CSF (*n* = 143), we yielded SUVR cutoff values for amyloid PET positivity with best discrimination of AD from the CN group, and then, the SUVR cutoffs were used to determine the cutoff values for CSF biomarkers following Youden’s index to differentiate participants with amyloid PET positivity (*n* = 128). Finally, we evaluated the predictive performances of CSF AD biomarkers in 139 subjects who provided CSF samples, using the amyloid PET-based cutoff values of CSF AD biomarkers

A total of 271 subjects out of 331 KBASE participants underwent amyloid imaging via ^11^C-PiB PET (*n* = 80) or ^18^F-flutemetamol PET (*n* = 191). We aligned PET images to the corresponding T_1_-weighted MRI and the standard uptake value ratio (SUVR) of each region of interest (ROI), which was obtained by dividing the mean uptake value for all voxels within the ROI by the mean value of the reference region. Composite SUVR values were calculated by averaging the SUVR values for the prefrontal, orbitofrontal, parietal, lateral temporal, anterior cingulate, and posterior cingulate/precuneus regions [24]. We determined the amyloid PET positivity based on cutoff values from the composite SUVR of cortical PiB retention to differentiate CN from AD, as described previously [25]. In the participants without lumbar puncture (*n* = 143) among 271 subjects with amyloid PET imaging, we yielded SUVR cutoff values for amyloid PET positivity with best discrimination of AD from the CN group, which were used to determine the cutoff values for CSF biomarkers following Youden’s index to differentiate participants with amyloid PET positivity (*n* = 128). Finally, we evaluated the predictive performances of CSF AD biomarkers in 139 subjects who provided CSF samples, using the cutoff values of CSF AD biomarkers determined by amyloid PET analysis (Fig. 1).

### Statistical analysis

Groups were compared using chi-square tests for categorical variables or the Kruskal–Wallis test followed by post hoc Dunn’s multiple comparisons for continuous variables. Receiver-operating characteristic curve (ROC) analyses were carried out to assess the diagnostic utility of various CSF biomarkers to distinguish AD from CN using Prism (v. 6.0; GraphPad Software, San Diego, CA, USA). Cutoffs with the highest agreement on Aβ-PET of CSF AD biomarkers using Youden’s rule were used to determine diagnostic performance and to assess the predictability of clinical progression. For the latter analysis, we compared the clinical scores of 139 participants who provided CSF and one or more annual follow-up assessments over 3 years between the normal and AD-like signature groups (i.e., above or below the cutoffs for each biomarker) using mixed-effect modeling. We further assessed significant time × group interaction effects for the ability of CSF biomarkers to predict change in cognitive decline of 139 total subjects, MCI group, or cognitively normal subjects (CN plus SCD), adjusting for covariates (age, gender, education years, and ApoE genotype) using analyses of covariance models (SPSS, ver. 19.0, Chicago, IL). To assess the correlation between immunoassays, we calculated the correlation coefficients, and we performed the Passing-Bablok regression which is suitable procedure for method comparison. The Passing-Bablok regression assumes an error for both axes (*x* and *y*) and is robust for method comparison in the presence of one or few outliers [26].

## Results

### Demographics and clinical characteristics

Median values for age and education years and frequency of ApoE e4 allele among the four groups (71 CN, 99 SCD, 89 MCI, and 72 AD) differed significantly, although there was no significant difference for these measures between CN and SCD. Scores on cognitive function and neuropsychological tests among the entire sample differed significantly between the four groups, with descending performance among CN>SCD>MCI>AD, as reported previously [25]. Clinical characteristics and neuropsychological test results among the subgroup of 139 participants who provided CSF (29 CN, 58 SCD, 29 MCI, and 23 AD) were consistent with the total sample of 331 subjects. Ages within the CN and SCD groups were similar, and younger than MCI and AD. Education years among the CN and MCI groups were comparable, and higher than SCD or AD. Gender distribution and ApoE e4 allele frequency differed significantly among the groups (Table 1 and Table S1).

**Table 1:** Clinical characteristics of 139 participants who provided CSF, according to the AD clinical spectrum

Characteristics	CN (*n* = 29)	SCD (*n* = 58)	MCI (n = 29)	AD (*n* = 23)	*P* value
Age, median (95% CI), y	63.0 (58–67)	66.5 (63–69)	72.0 (66–76) ^a,b^	71 (67–79) ^a,b^	< 0.001
Education, median (95% CI), y	12 (9–16)	6 (6–9) ^a^	10 (6–12)	6 (6–9) ^a,c^	< 0.001
Gender (M:F)	10: 19	29:29	21:8	6:17	< 0.004†
MMSE, median (95% CI)	29 (28–29)	26 (25–27) ^a^	24 (21–26) ^a^	16 (15–19) ^a,b,c^	< 0.001
SMCQ, median (95% CI)	1 (1–2)	5 (4–6) ^a^	5 (3–7) ^a^	8 (5–12) ^a,b^	< 0.001
SBT, median (95% CI)	0 (0–2)	4 (2–4) ^a^	7 (4–11) ^a^	18 (17–22) ^a,b,c^	< 0.001
CERAD, mean Z, median (95% CI)	0.48 (0.42–0.61)	0.14 (0.01–0.23)	−0.81 (− 1.13 – − 0.33) ^a,b^	−1.65 (− 2.02 – − 0.94) ^a,b^	< 0.001
BDS-ADL, median (95% CI)	0 (0–0)	0 (0–0.5) ^a^	0 (0–1) ^a^	2.5 (1–4.5) ^a,b,c^	< 0.001
ESS, median (95% CI)	5 (4–7)	4 (4–5)	3 (1–4)	2 (1–5)	0.0127
CDR 0:0.5:1	29:0:0	58:0:0	0:29:0	0:8:15	< 0.001†
CDR-SB, median (95% CI)	0 (0–0)	0 (0–0)	0.5 (0.5–1.0) ^a,b^	5 (3.5–6.0) ^a,b^	< 0.001
GDS, median (95% CI)	5 (3–8)	8 (7–12) ^a^	9 (5–15)	12 (7–20) ^a^	0.003
Aβ PET(+), % (−:+)	7.1 (26:2)	21.1 (41:11)	37.0 (17:10)	76.2 (5:16)	< 0.001†
ApoE ε4 carrier, % (−:+)	17.2 (24:5)	20.7 (46:12)	20.7 (23:6)	56.5 (10:13)	0.008†

*MMSE* Mini-Mental State Examination, *SMCQ* Subjective Memory Complaint Questionnaire, *SBT* Short Blessed Test, *CERAD* Consortium to Establish a Registry for AD, *BDS-ADL* Blessed Dementia Scale-Activities of Daily Living, *ESS* Epworth Sleepiness Scale, *CDR* Clinical Dementia Rating scale, *CDR-SB* CDR-Sum of Boxes, *GDS* Geriatric Depression Scale, *ApoE* apolipoprotein E

^a^*P* < 0.05 versus CN; ^b^*P* < 0.05 versus SCD; ^c^*P* < 0.05 versus MCI by Dunn’s multiple comparison following the Kruskal–Wallis test

†Chi-square test

Amyloid-PET positivity was determined by cutoff values for composite SUVR values in 143 subjects without CSFs. For ^11^C-PiB PET, the cutoff SUVR value was 1.15, with 100% sensitivity and 100% specificity to discriminate CN from AD participants following Youden’s rule. For ^18^F-flumetamol PET, the cutoff SUVR value was 0.64, with 85% sensitivity and 96% specificity to discriminate CN from AD participants. Four (7.1%) of 56 CN participants, 21 (23.9%) of 88 SCD participants, 29 of 73 participants with MCI (39.7%), and 42 (77.8%) of 54 patients with AD showed amyloid deposition. When we analyzed the subgroup who provided CSF (*n* = 139), the results were similar to those from the total sample, i.e., the percentages of amyloid PET-positivity for CN (*n* = 28), SCD (*n* = 52), MCI (*n* = 27), and AD (*n* = 21) were 7.1%, 21.1%, 37.0%, and 76.2%, respectively. In the AD group diagnosed by clinical evaluation, 3 patients were amyloid negative and showed normal CSF Aβ42 level; therefore, we excluded these patients from AD groups following the A/T/N criteria of biological definition of AD in further analysis.

### Cerebrospinal fluid biomarker levels

**Fig. 2:**
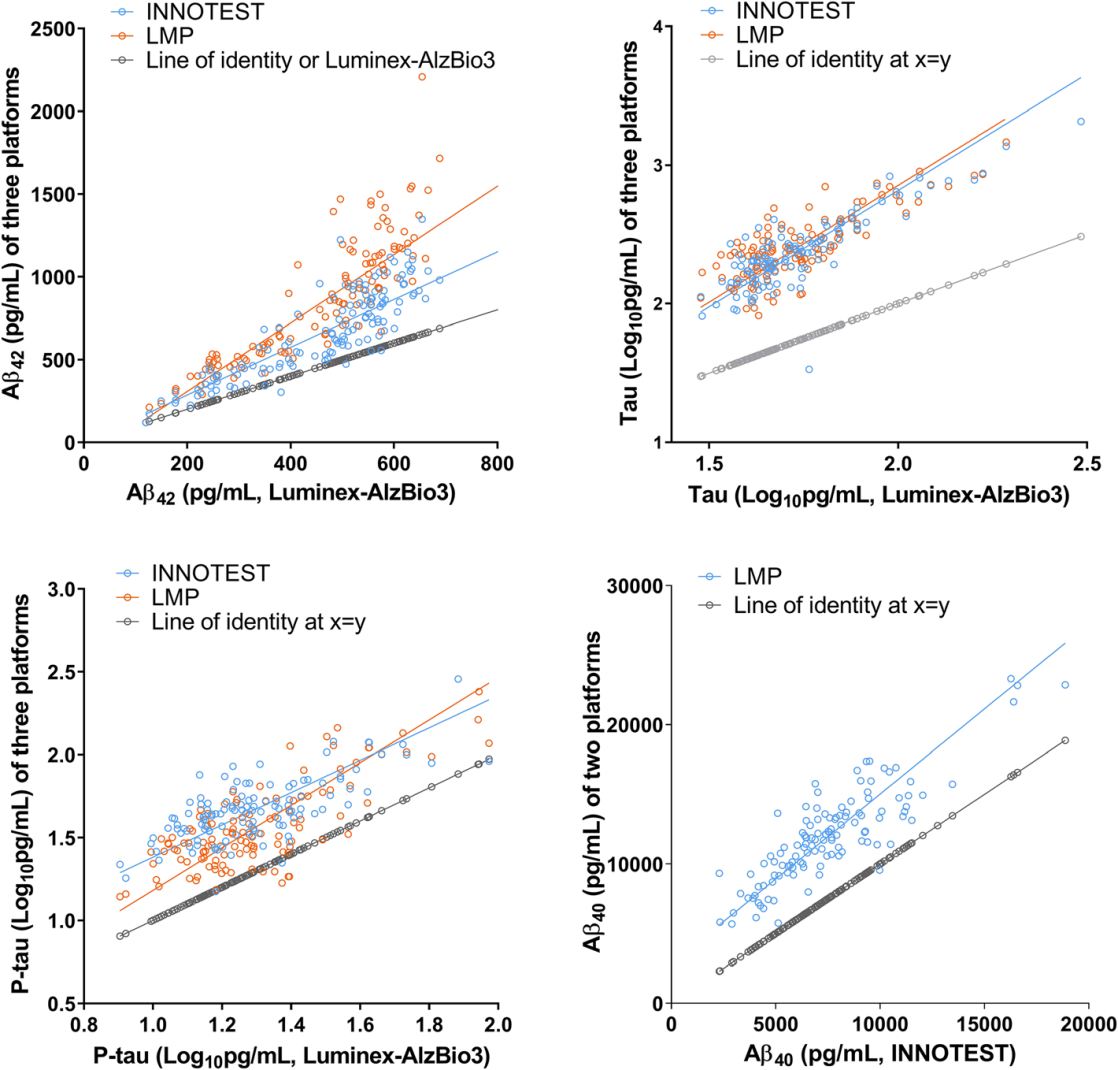
Correlations among CSF biomarker levels determined by three immunoassay platforms. The Spearman correlation coefficients between Aβ42, t-tau, and p-tau levels measured by Luminex and INNOTEST were 0.87, 0.76, and 0.71, respectively, and the coefficients between the Luminex and Lumipulse G were 0.90, 0.71, and 0.76, respectively. Aβ42, t-tau, p-tau, and Aβ40 levels measured by INNOTEST and Lumipulse G showed strong correlation coefficients, i.e., 0.91, 0.83, 0.91, and 0.86, respectively. Solid lines indicate the fitted lines of the Passing-Bablok regression. The Passing-Bablok regression analysis for method comparison showed that Aβ42 levels by Luminex were not valid in cusum test for linearity. For tau proteins among 3 assays or Aβ42 levels between INNOTEST and Lumipulse, the analysis showed the comparability between immunoassay methods. Gray symbols and lines indicated the values of *x* = *y*

The Aβ42 levels measured by 3 immunoassays in patients with MCI and AD were significantly lower than those in the CN group, while the levels among those with SCD were comparable to the CN group. The Aβ40 levels measured by ELISA and Lumipulse G did not differ among the groups. The levels of t-tau, p-tau, t-tau/Aβ42, and p-tau/Aβ42 determined by the three platforms in the AD group were significantly higher than those measured in the CN group. For those with MCI, the p-tau/Aβ42 levels measured by Lumipulse and Luminex were significantly higher than in the CN group. The ratio of t-tau/Aβ42 in the MCI group measured by Lumipulse was higher than in the CN group, while the levels in the MCI group measured by other assays were comparable with the CN group. In all immunoassay platforms, the mean Aβ42 level in the AD group was approximately 50% of the CN group level (Table 2 and supplementary figure 1), consistent with a previous study [27]. When we compared the ratio of Aβ42/Aβ40 measured by INNOTEST or Lumipulse G, the ratio among those with AD was significantly lower compared with the CN and SCD groups. The ratio in the MCI group measured by Lumipulse, but not by INNOTEST, was significantly lower than in the CN or SCD groups. As shown in Fig. 2, the biomarker levels from the various immunoassay platforms were strongly intercorrelated. For method comparison among immunoassays, we performed the Passing-Bablok regression analysis. The Passing-Bablok regression for tau levels showed the comparability among immunoassay methods. However, the regression was not valid for Aβ42 levels measured by Luminex since the cusum linearity test showed a nonlinearity in Aβ42 levels between Luminex and other immunoassays, which indicates the presence of a systematic difference and a proportional difference between Luminex and INNOTEST or Lumipulse.

**Table 2:** CSF biomarker levels measured by three immunoassay platforms

Groups	CSF biomarker levels, median (95% CI)						
	Aβ_42_	Aβ_40_	T-tau	P-tau	T-tau/Aβ_42_	P-tau/Aβ_42_	Aβ_42_/Aβ_40_
Luminex-AlzBio3							
CN	573.2 (511.3–600.6)	n.d.	46.1 (42.1–55.4)	16.1 (13.7–18.6)	0.09 (0.08–0.10)	0.03 (0.03–0.03)	n.d.
SCD	520.3 (484.2–570.5)	n.d.	46.0 (41.4–55.6)	16.9 (14.0–20.1)	0.09 (0.08–0.11)	0.03 (0.03–0.04)	n.d.
MCI	411.2 ^a,b^ (282.8–541.1)	n.d.	48.4 (42.0–63.0)	21.2 (15.1–25.5)	0.12 (0.08–0.18)	0.05 ^a^ (0.03–0.08)	n.d.
AD	258.9 ^a,b,c^ (234.0–305.0)	n.d.	81.6 ^a,b,c^ (67.4–127.2)	32.5 ^a,b^ (28.9–51.5)	0.36 ^a,b,c^ (0.24–0.57)	0.14 ^a,b,c^ (0.12–0.22)	n.d.
INNOTEST							
CN	756.7 (583.8–879.8)	6724 (5160–9070)	195.0 (156.1–239.7)	38.5 (35.0–49.7)	0.26 (0.23–0.32)	0.05 (0.05–0.07)	0.11 (0.09–0.12)
SCD	794.9 (642.0–950.7)	7405 (6388–9565)	199.5 (145.6–270.4)	43.8 (34.6–50.1)	0.26 (0.20–0.31)	0.05 (0.04–0.06)	0.10 (0.09–0.12)
MCI	565.1 ^b^ (390.4–734.9)	7158 (5551–8164)	207.7 (156.3–291.5)	46.4 (34.1–53.6)	0.31 (0.23–0.66)	0.07 (0.05–0.12)	0.09 (0.06–0.12)
AD	407.5 ^a,b,c^ (328.1–412.0)	6504 (5664–8218)	548.0 ^a,b,c^ (389.4–832.0)	72.5 ^a,b,c^ (56.9–113.8)	1.55 ^a,b,c^ (0.95–2.71)	0.24 ^a,b,c^ (0.14–0.38)	0.05 ^a,b,c^ (0.05–0.07)
Lumipulse							
CN	1104 (850.2–1340)	12,521 (10583–14,851)	224.0 (176.0–254.8)	28.7 (22.9–33.5)	0.20 (0.16–0.24)	0.03 (0.02–0.03)	0.09 (0.08–0.09)
SCD	958.9 (763.9–1148)	12,098 (10655–13,956)	221.5 (174.8–284.5)	28.3 (25.0–37.6)	0.21 (0.17–0.27)	0.03 (0.03–0.03)	0.09 (0.08–0.09)
MCI	651.4 ^a,b^ (465.1–884.6)	10,686 (9385–12,829)	253.0 (192.0–342.0)	35.0 (24.7–52.1)	0.29 ^a^ (0.22–0.83)	0.04 ^a,b^ (0.03–0.12)	0.07 ^a,b^ (0.04–0.09)
AD	497.9 ^a,b^ (407.6–527.7)	11,735 (9603–12,385)	544.0 ^a,b,c^ (448.0–735.8)	89.8 ^a,b,c^ (66.3–115.7)	1.35 ^a,b,c^ (4.03–1.63)	0.22 ^a,b,c^ (0.15–0.26)	0.04 ^a,b,c^ (0.04–0.05)

^a^*P* < 0.05 versus CN; ^b^*P* < 0.05 versus SCD; ^c^*P* < 0.05 versus MCI by Dunn’s multiple comparison following Kruskal–Wallis test

### Agreement on amyloid PET and CSF biomarker levels determined by three platforms

CSF amyloid positivity in the subgroup who provided CSF was determined using the cutoff of mean SUVR of amyloid PET from 143 participants (i.e., 1.15 for PiB and 0.64 for ^18^F-flutemetamol retention), which showed the highest discriminability between AD and CN.^21^ Based on the amyloid PET results, we determined CSF biomarker cutoffs at the highest agreement rate for amyloid deposition in the 128 participants who provided CSF and underwent the amyloid PET test. In all immunoassay platforms, Aβ42 (AUC = 0.857–0.897), t-tau/Aβ42 (AUC = 0.842–0.884), p-tau/Aβ42 (AUC = 0.840–0.892), and Aβ42/Aβ40 (AUC = 0.856–0.896) showed higher agreement than did t-tau (AUC = 0.694–0.803) or p-tau (AUC = 0.717–0.839), as expected (Table 3). ROC comparison analysis showed the comparable AUC levels for CSF Aβ42, t-tau/Aβ42, p-tau/Aβ42, or Aβ42/Aβ40 among immunoassays. Combination of biomarkers (i.e., ratios) did not show the higher AUC than Aβ42 alone for amyloid PET agreement in all platforms.

**Table 3:** ROC parameters for CSF biomarkers measured by different immunoassay platforms to discriminate participants with amyloid-PET positivity from those with amyloid-PET negativity

Assay platforms	Parameters	Aβ_42_	Aβ_40_	T-tau	P-tau	T-tau/Aβ_42_	P-tau/Aβ_42_	Aβ_42_/Aβ_40_
Luminex-AlzBio3	n*	125	–	124	125	124	125	
	ROC AUC	0.897	–	0.694	0.810	0.852	0.892	–
	Cut-off value	380.6 pg/mL	–	75.6 pg/mL	21.02 pg/mL	0.133	0.045	–
	PPA (%)	79.5	–	43.6	74.4	79.5	87.2	–
	NPA (%)	93.0	–	97.7	81.4	92.9	88.4	–
INNOTEST	*n*	123	123	115	109	115	109	123
	ROC AUC	0.860	n.s.	0.803	0.717	0.884	0.846	0.896
	Cut-off value	478.3 pg/mL	–	247.3 pg/mL	46.3 pg/mL	0.484	0.079	0.091
	PPA (%)	76.9	–	77.8	73.5	83.3	80.0	89.7
	NPA (%)	94.1	–	73.4	60.8	94.9	87.8	83.3
Lumipulse	*n*	123	126	123	123	123	123	123
	ROC AUC	0.857	n.s.	0.791	0.839	0.842	0.840	0.856
	Cut-off value	642.1 pg/mL	–	337 pg/mL	36.0 pg/mL	0.315	0.051	0.060
	PPA (%)	79.5	–	59.0	79.5	84.6	84.6	84.6
	NPA (%)	88.1	–	89.3	78.6	88.1	92.9	91.7

*ROC AUC* area under the receiver operating characteristic curve, *PPA* positive percent agreement, *NPA* negative percent agreement

*T-tau measured by Luminex of one sample and t-tau and p-tau measured using INNOTEST kits of 10 and 16 samples respectively were excluded following acceptance criteria of SOP, and two samples could not be measured due to loss of samples. 3 AD patients with Aβ-PET negative and normal CSF Aβ42 level were excluded to follow the A/T/N criteria for biological definition of AD

The ability to discriminate AD from CN in 139 participants using clinical-based diagnostic cutoffs is summarized in Table S2 (Additional file). The AUC of t-tau/Aβ42 (AUC = 0.913, 0.927, and 0.952 in Luminex, INNOTEST, and Lumipulse, respectively), p-tau/Aβ42 (AUC = 0.897, 0.912, and 0.946 in Luminex, INNOTEST, and Lumipulse, respectively), and Aβ42/Aβ40 (AUC = 0.922 and 0.952 in INNOTEST and Lumipulse, respectively) were higher than Aβ42 alone (AUC = 0.907, 0.876, and 0.889 in Luminex, INNOTEST, and Lumipulse, respectively), except for p-tau/Aβ42 versus Aβ42 in Luminex. When we compared PET-based cutoffs of Aβ42 to discriminate amyloid positive from negative patients (Table 3) with the clinical-based cut-off values with highest discrimination ability of AD from CN (Table S2), the cutoffs were comparable (i.e., 3~12% of difference). Aβ42 (AUC = 0.808, *P* < .0001), t-tau/Aβ42 (AUC = 0.765, *P* = .0008), p-tau/Aβ42 (AUC = 0.780, *P* = .0004), and Aβ42/Aβ40 (AUC = 0.769, *P* = .0006) from the Lumipulse assay showed significant discriminability between the MCI and CN groups, which showed low sensitivity (48.1–51.9%) but higher specificity (85.7–96.4%). In the other immunoassay platforms, the diagnostic performance for discrimination between MCI and CN groups was like that for Lumipulse. As expected, when we compared the CSF biomarker levels between amyloid PET-positive and PET-negative groups, all biomarkers determined by the three assay platforms except Aβ40 differed significantly (Additional file: Table S3). In all assay platforms, the Aβ42 level in amyloid PET-positive participants was 51.8–54.5% of the amyloid PET-negative group.

### Performance of CSF biomarkers for predicting clinical progression

To test clinical predictability of the baseline PET-based cutoffs for cognitive decline, we followed participants up to 3 years. When we compared the progressive decline of cognitive function between above or below the CSF biomarker cutoffs, groups with AD-like CSF biomarker signature (i.e., lower Aβ42 or Aβ42/Aβ40, and higher p-tau/Aβ42 or t-tau/Aβ42 than cutoffs) showed more rapid decline in cognitive function (e.g., CDR, CDR-SB, MMSE, BDS-ADL, construction praxis, clock drawing, and Short Blessed Test scores) compared with the groups with normal CSF biomarker signatures (*P* < .05). This significant difference remained after adjusting for either age and ApoE genotype or age, ApoE genotype, gender, and education years (Table 4). In the MCI group, although we observed a trend toward more rapid MMSE score decline in the group with an AD-like CSF signature (*n* = 14) compared with the group with a normal CSF signature (*n* = 13), the difference in the progressive cognitive decline did not reach statistical significance (Additional file: Supplementary Figure 2). A more rapid increase in CDR-SB scores among those in the MCI group with a higher t-tau/Aβ42, higher p-tau/Aβ42, or lower Aβ42/Aβ40 than among those in the MCI group with a normal CSF signature was observed, though the difference was not statistically significant. During follow-up, we observed that in the groups with normal cognition or without significant cognitive dysfunction (i.e., CN plus SCD, *n* = 80), there was more rapid progression of CDR scores (*F* = 3.109, *P* = .032) in the group with low Aβ42, more rapid progression of MMSE scores (*F* = 3.405, *P* = .023) in the group with high t-tau/Aβ42, and more rapid progression of construction recall (*F* = 3.432, *P* = .022) in the group with higher t-tau. When we analyzed AD patients whose diagnosis were determined clinically, the more rapid progression of CDR scores (*F* = 3.109, *P* = 0.032), CDR-SB scores (*F* = 8.543, *P* = 0.003), and geriatric depression scores (*F* = 4.942, *P* = 0.018) was observed in the group with low Aβ42 (< 642.1 pg/mL), as compared to the group with normal Aβ42 level. In AD patients with high t-tau level (> 337 pg/mL), the more rapid progression of CDR-SB scores (*F* = 2.887, *P* = 0.0431) was observed.

**Table 4:** Predictive performance of CSF biomarkers for clinical progression among 139 samples

Clinical variables	Biomarkers					
	Aβ42	T-tau	P-tau	Aβ42/Aβ40	t-tau/Aβ42	p-tau/Aβ42
	*F*, *P* value					
CDR	**4.139, 0.008**	1.512, 0.215	1.127, 0.341	**3.627, 0.015**	**2.831, 0.041**	**3.639, 0.015**
CDR-SB	**8.308, 0.0001**	1.584, 0.197	1.011, 0.390	**5.982, 0.001**	**4.472, 0.005**	**6.416, 0.0001**
BDS-ADL	3.471, 0.018	1.634, 0.185	0.749, 0.525	**2.975, 0.034**	2.199, 0.092	**3.095, 0.030**
SBT	1.566, 0.201	1.500, 0.218	0.661, 0.578	2.002, 0.117	2.365, 0.074	1.720, 0.167
MMSE	**3.434, 0.020**	2.362, 0.076	1.358, 0.260	**4.227, 0.007**	**4.277, 0.007**	**5.030, 0.003**
Constructional praxis	**2.921, 0.038**	1.596, 0.195	0.335, 0.800	2.071, 0.109	2.244, 0.088	2.088, 0.107
Clock drawing	**3.295, 0.024**	**3.482, 0.019**	1.356, 0.261	**4.751, 0.004**	**5.091, 0.003**	**5.034, 0.003**

Ten subjects with AD-like CSF signature (2, 1, and 7 at first, second, and third follow-ups, respectively) and 9 subjects with normal CSF signature (1, 4, and 4 at third, second, and first follow-ups, respectively) were lost to follow-up. Numbers in bold indicate the variables with significance under ANCOVA analysis

*CDR* Clinical Dementia Rating scale, *CDR-SB* CDR-Sum of Boxes, *BDS-ADL* Blessed Dementia Scale-Activities of Daily Living, *SBT* Short Blessed Test, *MMSE* Mini-Mental State Examination

## Discussion

While AD diagnosis is largely based on clinical and neuropsychological test performance and cognitive function, postmortem diagnosis based on autopsy has shown that ~ 30% of AD cases are misdiagnosed [28]. Given that CSF immunoassays and amyloid PET analyses have shown promise as biomarkers reflecting the trajectory of AD pathology [29], amyloid PET analysis has been widely accepted for its strong agreement with pathological amyloid aggregates [30, 31], and increasing diagnostic confidence [32, 33]. In addition, the use of amyloid-PET as the standard for establishing CSF AD biomarker cutoffs may reduce inter-center variability compared with clinical-based cutoffs [34] and may be useful for identifying AD pathology antemortem [14]. Therefore, we determined CSF biomarker cutoffs for evaluating diagnostic and predictive performance based on the best levels of agreement with amyloid PET status. When we compared agreement among CSF biomarkers with PET results using three immunoassay platforms, Aβ42, t-tau/Aβ42, p-tau/Aβ42, and Aβ42/Aβ40 showed a higher overall agreement than did t-tau or p-tau alone. For the diagnostic performance of the ratios, compared with Aβ42, t-tau or p-tau alone, using the PET-based cutoffs was clearly better. The agreement performance of CSF amyloid signature with amyloid PET positivity is comparable among immunoassay platforms (Table 3). In addition, we observed strong correlations among the biomarker levels determined by the three immunoassay platforms (Fig. 2). These results indicate that CSF biomarkers measured by a novel Lumipulse automated immunoassay with CRM-based method validation provide a more accessible, antemortem alternative to evaluating patients underlying AD pathophysiology, and an opportunity to discriminate symptomatic AD patients in the clinic or in AD trials.

Compared with previous studies [20, 35–37], cutoffs of Aβ42 and t-tau by INNOTEST or Luminex-AlzBio3 for AD diagnosis were higher and lower, respectively, leading to a lower t-tau/Aβ42 ratio cutoff herein. Compared with the t-tau or p-tau cutoffs from INNOTEST-based diagnostics reported in another Korean population, ours were lower [38]. These cutoff discrepancies for Aβ42, t-tau, or p-tau may have been caused by either inter-laboratory variability in determining the CSF AD biomarker levels using a manual immunoassay, assay concepts, or preanalytical variables. Significant intra-laboratory analytical variability in the levels of Aβ42, t-tau and p-tau from Luminex-AlzBio3 is unlikely, as we observed similar biomarkers levels between the KBASE-V cohort and another small, independent cohort (Additional files: Supplementary Method 3 and Table S4) and a low between-run %CV. Considering the fully automated Lumipulse immunoassay, a previous study reported higher cutoffs for Aβ42, t-tau, and t-tau/Aβ42 (approximately 0.54) for discriminating amyloid PET positivity compared with our study [14]. In another study, cutoffs for Aβ42, t-tau, and p-tau and their ratios were higher than herein [39]. However, another study reported lower diagnostic cutoffs for Aβ42; higher cutoffs for p-tau, p-tau/Aβ42 (0.086), and t-tau/Aβ42 (0.578); and a similar cutoff for t-tau compared with our amyloid-positive results herein [23]. Those previous studies showed an approximately 0.53–0.62 t-tau/Aβ42 for discriminating amyloid PET positivity or AD diagnosis, which is approximately twofold higher than our study cutoff of t-tau/Aβ42 (0.315). However, those investigators reported a similar Aβ42/Aβ40 cutoff (~ 0.06), indicating that the Aβ42/Aβ40 ratio may be more reliable for AD diagnosis than a single biomarker or other ratios. Given that fully automated immunoassay has low analytical variability in the CRM-adjusted CSF AD biomarker levels compared with manual immunoassay [14], comparing the discrepancy in the Aβ42 level or tau proteins measured by Lumipulse in our study with other studies may be due to clinical variables or pre-analytics. Recently, racial disparity has been identified in CSF tau-based biomarker levels in both patients with MCI and community-living older adults, which remained after covariate-adjusted analysis [40, 41]. Although CSF biomarkers in these studies were measured via manual assays rather than fully automated, their reported tau protein levels reflected interethnic differences (i.e., lower levels among African-American compared with white participants). To our knowledge, ours are the first CSF AD biomarker data from a Korean population which were measured by both manual and fully automated immunoassay systems. In the future, direct comparison of CSF AD biomarkers across different AD disease continuums for race-specific AD diagnostic cutoffs, or evaluation of amyloid PET agreement among different races, including Korean, will clarify possible interethnic differences in CSF AD biomarker levels. Other possible influences on CSF AD biomarker levels may be different covariates, including different levels of mixed pathologies, comorbidities, or ages [42, 43].

Regardless of differing CSF AD biomarkers cutoffs between immunoassay platforms, amyloid PET-based cutoffs of biomarkers showed significant diagnostic performance for discriminating between AD and CN groups in all immunoassay platforms. Furthermore, using PET-based cutoffs, we observed that Aβ42 and ratios (i.e., Aβ42/Aβ40, t-tau/Aβ42, and p-tau/Aβ42) measured by Lumipulse predicted progression of cognitive decline and deterioration of daily living over 3 years in our subgroup of 139 participants, after adjusting for covariates. Despite our relatively small sample, the significance of these results is due in part to this being the first report of the clinical predictability of amyloid PET-based cutoffs for CSF AD biomarkers, determined by a fully automated immunoassay in a multicenter Korean cohort. Although the predictability of cognitive decline in CSF biomarkers has been reported in a previous study [44], when considering the possible confounding effects of clinical variables and racial disparity on diagnostic and/or predictive performance of CSF AD biomarker cutoffs, further studies with larger, more diverse samples from different races are warranted. The predictability of these CSF biomarkers for clinical progression was not significant among those with MCI. We observed a trend toward different clinical trajectories between the MCI group with an AD-like CSF signature and those with normal levels; thus, this may have been due to our relatively small number of participants with MCI. Although the number of participants without significant cognitive dysfunction (CN plus SCD) was small, some biomarkers, Aβ42, t-tau/Aβ42, and t-tau, may predict cognitive decline. In AD patients, although low Aβ predicted the more rapid cognitive decline, number of AD patients with normal CSF Aβ level was quite low (*n* = 4) and 3 out of 4 AD patients with normal CSF Aβ42 showed normal Aβ-PET as well; hence, these result finding should be replicated in other cohorts with larger sample sizes.

### Limitation

This study’s limitations include its relatively small number of participants on whom CSF analyses were performed, which may have resulted in failure to detect statistical significance in subgroup analyses. Despite this, it is important to note the clinical utility of these findings. To our knowledge, ours is the first report of the significant clinical utility of CSF AD biomarkers simultaneously measured by both manual and fully automatic immunoassay platforms using Aβ PET status-based cutoffs in Korean. Although the clinical utility of immunoassays of CSF biomarkers is well-known, interethnic differences in CSF AD biomarker levels are possible which remains to be elucidated. Therefore, our results may be specific to Koreans, which warrants future comparisons using a fully automated immunoassay among different races. Another limitation is that the diagnostic performance of the amyloid PET-based cutoffs of CSF biomarkers in our study may have a risk of overfitting. We observed the excellent diagnostic performance of the amyloid PET-based cutoffs of CSF biomarkers measured by Luminex in another independent small cohort (Supplementary Table S4); however, since SUVR cut-offs for amyloid PET positivity was based on clinical diagnosis, the possibility of overfitting during determination and application of amyloid PET-based CSF cutoffs should be elucidated in the larger independent Korean cohort using an automated immunoassay platform.

## Conclusion

Despite limitations, our study demonstrates the clinical utility of Aβ PET-based cutoffs of CSF AD biomarkers in Koreans for the first time using a fully automated immunoassay, which agrees with manual immunoassays. Although it remains to be determined whether CSF AD biomarker levels and diagnostic cutoffs differ among various ethnic groups [40, 41], our study indicates that a fully automated immunoassay with minimal inter-laboratory variability can replace manual immunoassays to differentiate AD from CN populations, incorporate the framework’s amyloid/tau/neurodegeneration classification scheme for AD, and predict clinical progression. Regarding accelerating AD trials for developing disease-modifying drugs through multinational studies, the use of automated CSF AD biomarker measurements and determination of CSF cutoffs in different races will be vital.

## Supplementary Information

**Additional file 1.**

## Data Availability

The data reported in this manuscript are available within the article and/or its supplementary data. Additional data will be shared upon request by a corresponding author.

## References

[1] McKhann GM, Knopman DS, Chertkow H, Hyman BT, Jack CR, Kawas CH (2011). The diagnosis of dementia due to Alzheimer’s disease: recommendations from the National Institute on Aging-Alzheimer's Association workgroups on diagnostic guidelines for Alzheimer's disease. Alzheimers Dement.

[2] Blennow K, Zetterberg H (2018). Biomarkers for Alzheimer’s disease: current status and prospects for the future. J Intern Med.

[3] Olsson A, Vanderstichele H, Andreasen N, De Meyer G, Wallin A, Holmberg B (2005). Simultaneous measurement of beta-amyloid(1-42), total tau, and phosphorylated tau (Thr181) in cerebrospinal fluid by the xMAP technology. Clin Chem.

[4] Kang JH, Korecka M, Toledo JB, Trojanowski JQ, Shaw LM (2013). Clinical utility and analytical challenges in measurement of cerebrospinal fluid amyloid-beta(1-42) and tau proteins as Alzheimer disease biomarkers. Clin Chem.

[5] Irwin DJ, McMillan CT, Toledo JB, Arnold SE, Shaw LM, Wang LS (2012). Comparison of cerebrospinal fluid levels of tau and Abeta 1-42 in Alzheimer disease and frontotemporal degeneration using 2 analytical platforms. Arch Neurol.

[6] Fagan AM, Shaw LM, Xiong C, Vanderstichele H, Mintun MA, Trojanowski JQ (2011). Comparison of analytical platforms for cerebrospinal fluid measures of beta-amyloid 1-42, total tau, and p-tau181 for identifying Alzheimer disease amyloid plaque pathology. Arch Neurol.

[7] Shaw LM, Vanderstichele H, Knapik-Czajka M, Figurski M, Coart E, Blennow K (2011). Qualification of the analytical and clinical performance of CSF biomarker analyses in ADNI. Acta Neuropathol.

[8] Mattsson N, Andreasson U, Persson S, Arai H, Batish SD, Bernardini S (2011). The Alzheimer’s Association external quality control program for cerebrospinal fluid biomarkers. Alzheimers Dement.

[9] Mattsson N, Andreasson U, Persson S, Carrillo MC, Collins S, Chalbot S (2013). CSF biomarker variability in the Alzheimer’s Association quality control program. Alzheimers Dement.

[10] Hansson O, Mikulskis A, Fagan AM, Teunissen C, Zetterberg H, Vanderstichele H (2018). The impact of preanalytical variables on measuring cerebrospinal fluid biomarkers for Alzheimer’s disease diagnosis: a review. Alzheimers Dement.

[11] Vandijck M, Kuwano R, Waligorska T, De Smet S, Tsukie T, Verdoodt L (2013). P1-166: inter-laboratory variation when using a unified test procedure for INNO-BIA AlzBio3. Alzheimers Dement.

[12] Bjerke M, Andreasson U, Kuhlmann J, Portelius E, Pannee J, Lewczuk P (2016). Assessing the commutability of reference material formats for the harmonization of amyloid-beta measurements. Clin Chem Lab Med.

[13] Bittner T, Zetterberg H, Teunissen CE, Ostlund RE, Militello M, Andreasson U (2016). Technical performance of a novel, fully automated electrochemiluminescence immunoassay for the quantitation of beta-amyloid (1-42) in human cerebrospinal fluid. Alzheimers Dement.

[14] Kaplow J, Vandijck M, Gray J, Kanekiyo M, Huyck E, Traynham CJ (2020). Concordance of Lumipulse cerebrospinal fluid t-tau/Abeta42 ratio with amyloid PET status. Alzheimers Dement.

[15] Janelidze S, Pannee J, Mikulskis A, Chiao P, Zetterberg H, Blennow K (2017). Concordance between different amyloid immunoassays and visual amyloid positron emission tomographic assessment. JAMA Neurol..

[16] Hansson O, Seibyl J, Stomrud E, Zetterberg H, Trojanowski JQ, Bittner T (2018). CSF biomarkers of Alzheimer’s disease concord with amyloid-beta PET and predict clinical progression: a study of fully automated immunoassays in BioFINDER and ADNI cohorts. Alzheimers Dement.

[17] Lee DY, Kim EH (2019). Therapeutic effects of amino acids in liver diseases: current studies and future perspectives. J Cancer Prev.

[18] Choi SH, Jung YK, Jang JA, Han S (2019). Idiopathic pulmonary arterial hypertension associated with a novel frameshift mutation in the bone morphogenetic protein receptor II gene and enhanced bone morphogenetic protein signaling: a case report. Medicine (Baltimore).

[19] Youn JC, Kim KW, Lee DY, Jhoo JH, Lee SB, Park JH (2009). Development of the subjective memory complaints questionnaire. Dement Geriatr Cogn Disord.

[20] Shaw LM, Vanderstichele H, Knapik-Czajka M, Clark CM, Aisen PS, Petersen RC (2009). Cerebrospinal fluid biomarker signature in Alzheimer’s disease neuroimaging initiative subjects. Ann Neurol.

[21] Agnello L, Piccoli T, Vidali M, Cuffaro L, Lo Sasso B, Iacolino G (2020). Diagnostic accuracy of cerebrospinal fluid biomarkers measured by chemiluminescent enzyme immunoassay for Alzheimer disease diagnosis. Scand J Clin Lab Invest.

[22] Bayart JL, Hanseeuw B, Ivanoiu A, van Pesch V (2019). Analytical and clinical performances of the automated Lumipulse cerebrospinal fluid Abeta42 and T-Tau assays for Alzheimer’s disease diagnosis. J Neurol.

[23] Leitao MJ, Silva-Spinola A, Santana I, Olmedo V, Nadal A, Le Bastard N (2019). Clinical validation of the Lumipulse G cerebrospinal fluid assays for routine diagnosis of Alzheimer’s disease. Alzheimers Res Ther.

[24] Jack CR, Lowe VJ, Senjem ML, Weigand SD, Kemp BJ, Shiung MM (2008). 11C PiB and structural MRI provide complementary information in imaging of Alzheimer’s disease and amnestic mild cognitive impairment. Brain..

[25] Hwang J, Jeong JH, Yoon SJ, Park KW, Kim EJ, Yoon B, et al. Clinical and biomarker characteristics according to clinical spectrum of Alzheimer’s disease (AD) in the validation cohort of Korean Brain Aging Study for the Early Diagnosis and Prediction of AD. J Clin Med. 2019;8(3):341. 10.3390/jcm8030341 PMC6463169 30862124

[26] Passing H (1983). Bablok. A new biometrical procedure for testing the equality of measurements from two different analytical methods. Application of linear regression procedures for method comparison studies in clinical chemistry, part I. J Clin Chem Clin Biochem.

[27] Kang JH, Vanderstichele H, Trojanowski JQ, Shaw LM (2012). Simultaneous analysis of cerebrospinal fluid biomarkers using microsphere-based xMAP multiplex technology for early detection of Alzheimer’s disease. Methods..

[28] Neuropathology Group. Medical Research Council Cognitive F, Aging S (2001). Pathological correlates of late-onset dementia in a multicentre, community-based population in England and Wales. Neuropathology Group of the Medical Research Council Cognitive Function and Ageing Study (MRC CFAS). Lancet.

[29] Blennow K, Mattsson N, Scholl M, Hansson O, Zetterberg H (2015). Amyloid biomarkers in Alzheimer’s disease. Trends Pharmacol Sci.

[30] Ossenkoppele R, Jansen WJ, Rabinovici GD, Knol DL, van der Flier WM, van Berckel BN (2015). Prevalence of amyloid PET positivity in dementia syndromes: a meta-analysis. JAMA..

[31] Klunk WE (2011). Amyloid imaging as a biomarker for cerebral beta-amyloidosis and risk prediction for Alzheimer dementia. Neurobiol Aging.

[32] Sanchez-Juan P, Ghosh PM, Hagen J, Gesierich B, Henry M, Grinberg LT (2014). Practical utility of amyloid and FDG-PET in an academic dementia center. Neurology..

[33] Ossenkoppele R, Prins ND, Pijnenburg YA, Lemstra AW, van der Flier WM, Adriaanse SF (2013). Impact of molecular imaging on the diagnostic process in a memory clinic. Alzheimers Dement.

[34] Zwan MD, Rinne JO, Hasselbalch SG, Nordberg A, Lleo A, Herukka SK (2016). Use of amyloid-PET to determine cutpoints for CSF markers: a multicenter study. Neurology..

[35] Ohkuma T, Ninomiya T, Tomiyama H, Kario K, Hoshide S, Kita Y (2017). Brachial-ankle pulse wave velocity and the risk prediction of cardiovascular disease: an individual participant data meta-analysis. Hypertension..

[36] Mulder C, Verwey NA, van der Flier WM, Bouwman FH, Kok A, van Elk EJ (2010). Amyloid-beta(1-42), total tau, and phosphorylated tau as cerebrospinal fluid biomarkers for the diagnosis of Alzheimer disease. Clin Chem.

[37] Mattsson N, Zetterberg H, Hansson O, Andreasen N, Parnetti L, Jonsson M (2009). CSF biomarkers and incipient Alzheimer disease in patients with mild cognitive impairment. JAMA..

[38] Park SA, Chae WS, Kim HJ, Shin HS, Kim S, Im JY (2017). Cerebrospinal fluid biomarkers for the diagnosis of Alzheimer disease in South Korea. Alzheimer Dis Assoc Disord.

[39] Alcolea D, Pegueroles J, Munoz L, Camacho V, Lopez-Mora D, Fernandez-Leon A (2019). Agreement of amyloid PET and CSF biomarkers for Alzheimer’s disease on Lumipulse. Ann Clin Transl Neurol.

[40] Morris JC, Schindler SE, McCue LM, Moulder KL, Benzinger TLS, Cruchaga C (2019). Assessment of racial disparities in biomarkers for Alzheimer disease. JAMA Neurol.

[41] Garrett SL, McDaniel D, Obideen M, Trammell AR, Shaw LM, Goldstein FC (2019). Racial disparity in cerebrospinal fluid amyloid and tau biomarkers and associated cutoffs for mild cognitive impairment. JAMA Netw Open.

[42] Wolfsgruber S, Molinuevo JL, Wagner M, Teunissen CE, Rami L, Coll-Padros N (2019). Prevalence of abnormal Alzheimer’s disease biomarkers in patients with subjective cognitive decline: cross-sectional comparison of three European memory clinic samples. Alzheimers Res Ther.

[43] Galasko D, Chang L, Motter R, Clark CM, Kaye J, Knopman D (1998). High cerebrospinal fluid tau and low amyloid beta42 levels in the clinical diagnosis of Alzheimer disease and relation to apolipoprotein E genotype. Arch Neurol.

[44] Tijms BM, Bertens D, Slot RE, Gouw AA, Teunissen CE, Scheltens P (2017). Low normal cerebrospinal fluid Abeta42 levels predict clinical progression in nondemented subjects. Ann Neurol.

