# Supplementary material for: Alzheimer’s cerebrospinal biomarkers from Lumipulse fully automated immunoassay: concordance with amyloid-beta PET and manual immunoassay in Koreans: CSF AD biomarkers measured by Lumipulse in Koreans
Source: Alzheimers Res Ther. 2021 Jan 12;13:22. doi: 10.1186/s13195-020-00767-3 (PMC7802266; doi:10.1186/s13195-020-00767-3)
Supplement: Supplementary file 1 — Additional file 1. [file 13195_2020_767_MOESM1_ESM.docx]

**Supplementary Information**

**Supplementary Method 1. Clinical diagnosis and exclusion criteria**

The cognitively normal (CN) group and those with subjective cognitive decline (SCD) showed normal performance on the memory tests of the Korean version of the Consortium to Establish a Registry for Alzheimer’s Disease (CERAD-K) after adjustment for age, gender, education, and global CDR scores of zero. However, as the AD clinical spectrum includes SCD, MCI, and AD, and SCD without impaired performance on cognitive tests represents the first symptomatic manifestation of AD, we divided cognitively unimpaired participants into CN and SCD. Enrollment of SCD participants followed the SCD criteria by the SCD-Initiative Working Group. In details, SCD subjects reported self-experienced persistent decline in cognitive function that are not related to an acute event in comparison with their previously normal status. They reported the symptoms to the clinician during the interview with a 10-item subjective cognitive decline questionnaire. SCD subjects showed age-, gender- and education-adjusted performance on standardized cognitive tests, which are used to classify MCI. When the cognitive decline in SCD can be explained by a psychiatric or neurologic disease, medical disorder, medication or substance use, we excluded the subjects. MCI diagnosis was based on the clinical criteria of MCI due to AD by the NIA-AA workgroups and modified from those proposed by Petersen et al., the *Diagnostic and Statistical Manual of Mental Disorder,* 4^th^ Edition (DSM-IV-TR), the criteria for probable AD according to the NIA-AA core clinical criteria and CDR score of 0.5 or 1. Exclusion criteria for all participants were: 1) presence of major psychiatric illness; 2) significant neurological or medical condition or comorbidities that could affect cognitive functioning; 3) contraindications for MRI scan (e.g., pacemaker, claustrophobia); 4) illiteracy; 5) significant visual or hearing difficulty, severe communication or behavioral problems that would make a clinical examination or brain scan difficult; 6) taking an investigational drug; and 7) pregnant or breastfeeding.

**Supplementary Method 2. CSF biomarker measurement by ELISA and Luminex platform**

Using one CSF aliquot, Aβ42, t-tau and p-tau were simultaneously measured using the Luminex manual multiplex immunoassay with the INNO-BIA AlzBio3 kit (Fujirebio Europe, Gent, Belgium) as previously described. Additional CSF aliquots were shipped and measured at a laboratory of Fujirebio N.V. using the INNOTEST manual assay (Aβ42, Aβ40, t-tau and p-tau) following the manufacturer’s instruction. All of the runs followed the SOP. For Luminex assay, acceptance criteria include enough bead count (>20) and low %CV (<25%) in duplicates of calibrators, aqueous buffer quality controls and CSFs (n=8). The average between-run coefficient of variations (%CV) of t-tau, Aβ42, and p-tau for calibrators (0.3–8.9%), aqueous controls (3.3–5.3%) and eight CSFs (2.3–19.3%) through five runs of Luminex assay were acceptable.

**Supplementary Method 3. Measurement of CSF biomarkers of an independent cohort using Luminex platform**

To confirm the diagnostic performance of Aβ PET-based predetermined cutoffs of CSF biomarkers measured by Luminex-xMAP, we applied the cutoffs to an independent cohort. Total of 34 subjects from 2 clinical centers (10 healthy controls, 7 MCI and 17 AD) were enrolled. The procedures of CSF collection, sample processing, and diagnostic procedures were consistent with the KBASE-V protocol. Healthy controls (HC) showed normal cognitive function without neuropsychiatric disorders. Fifteen out of 17 AD patients (88.2%), 3 out of 7 MCI patients (42.9%) and 1 out of 10 HC (10%) showed amyloid positivity in Aβ-PET analysis.


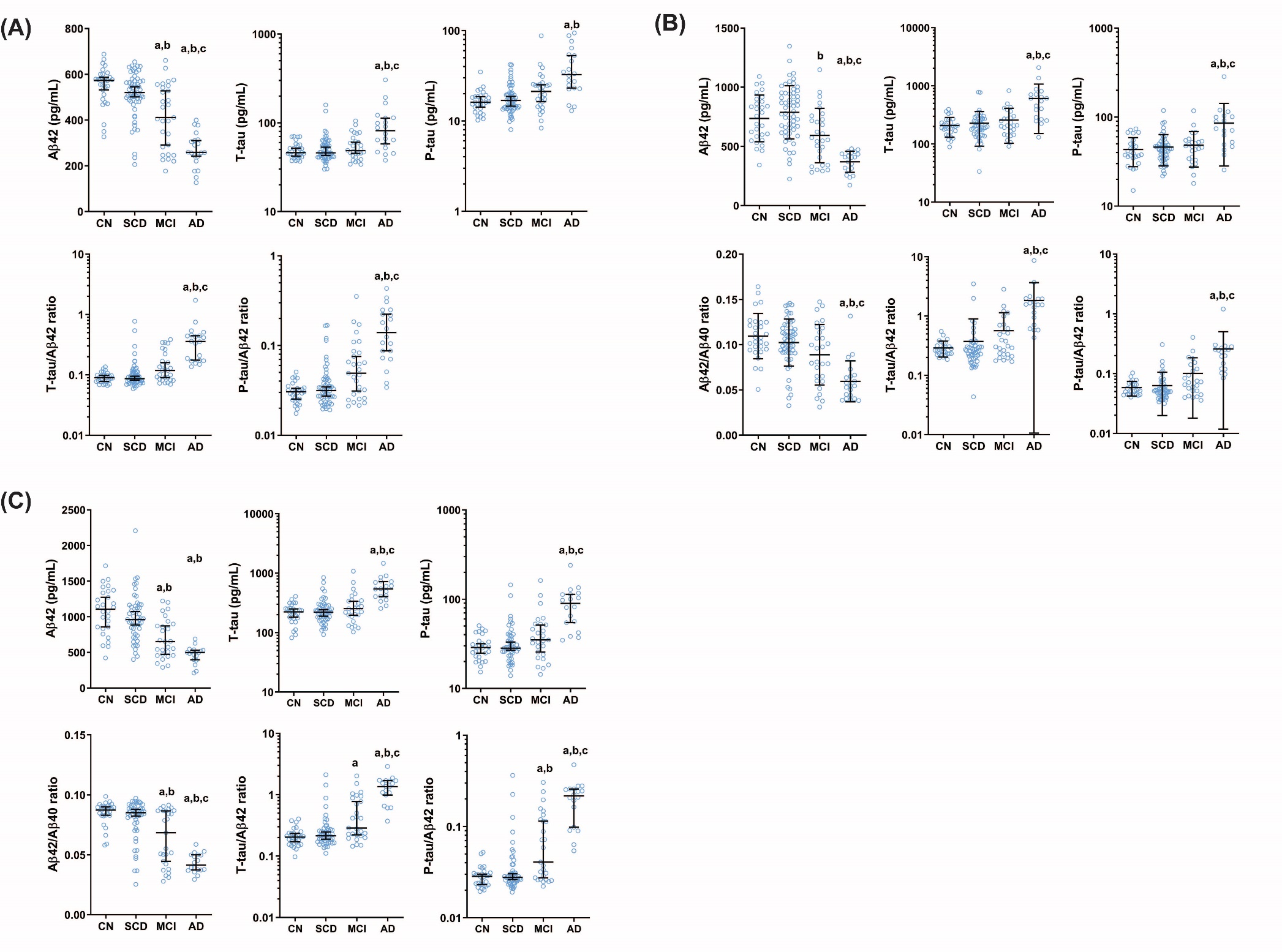


**Supplementary Figure 1. Scatter plots of CSF biomarker levels according to the diagnosis.**

CSF biomarkers were measured by (A) Luminex-AlzBio3, (B) INNOTEST ELISA kit, or (C) Lumipulse automated immunoassay. Horizontal and vertical bars indicate median and 95% confidence interval, respectively, and a, b, c indicate the significant difference as compared to CN, SCD, and MCI, respectively (Kruskal-Wallis test with Dunn’s multiple comparison).


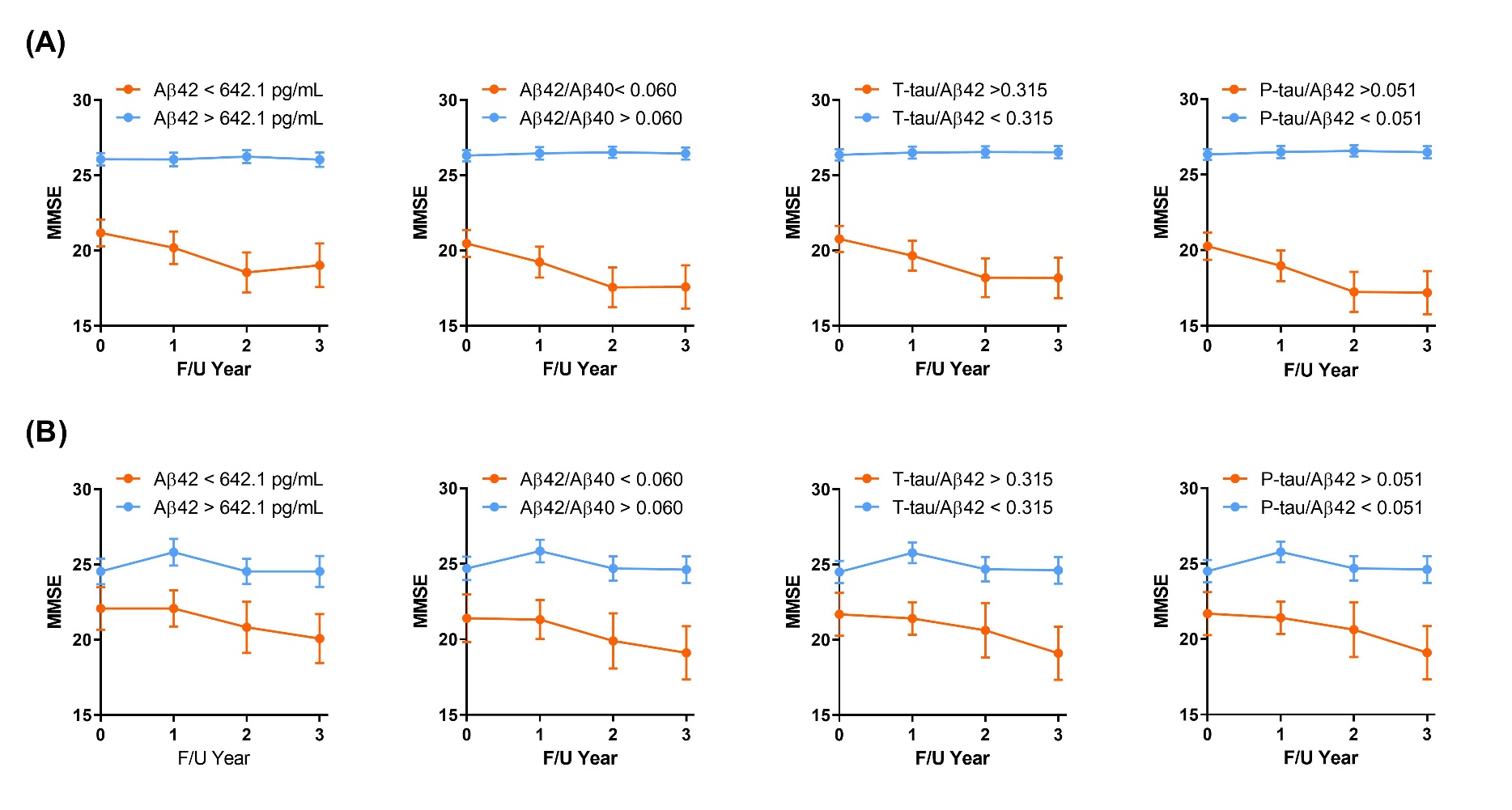


**Supplementary Figure 2. Progressive Changes in MMSE Scores in the Groups with Normal or AD-Like CSF Biomarker Signatures.**

Changes in mean MMSE scores over 3 years in (A) total of 139 subjects and (B) a subgroup of 27 MCI patients were shown. Vertical bars indicate standard errors of mean.

**Table S1**. **Neuropsychological Test Results According to the AD Clinical Spectrum in a Subgroup of 139 Participants with CSF Analyses**

| **Neuropsychological test** | **CN (n = 29)** | **SCD (n = 58)** | **MCI (n = 29)** | **AD (n = 23)** | **P value** |
| --- | --- | --- | --- | --- | --- |
| Auditory attention span, median (95% CI) | | | | | |
| Digit Span Forward | 8 (7 – 10) | 7 (6 – 8) | 7 (6 – 8) | 6 (4 – 7) ^a^ | < 0.001 |
| Digit Span Backward | 6 (5 – 7) | 4 (3 – 4) ^a^ | 4 (3 – 5) ^a^ | 3 (1 – 5) ^a^ | < 0.001 |
| Language, median (95% CI) | | | | | |
| Verbal Fluency, Z score | 0.26 (-0.24 – 0.73) | -0.32 (-0.52 – 0.20) | -0.35 (-0.89 – 0.25) | -1.34 (-1.80 – -0.97) ^a,b,c^ | < 0.001 |
| Boston Naming, Z score | 0.89 (0.52 – 1.28) | 0.58 (0.42 – 0.65) | 0.08 (-0.36 – 0.82) ^a^ | 0.24 (-1.58 – 0.44) ^a,b^ | < 0.001 |
| Episodic memory, median (95% CI) | | | | | |
| Word List Immediate Recall, Z score | 0.61 (0.35 – 1.03) | 0.26 (-0.03 – 0.43) | -0.94 (-1.18 – -0.13) ^a,b^ | -1.69 (-2.57 – -1.26) ^a,b,^ | < 0.001 |
| Word List Delayed Recall, Z score | 0.40 (0.16 – 0.88) | -0.05 (-0.37 – 0.30) | -1.44 (-1.79 – -0.89) ^a,b^ | -2.47 (-2.84 – -2.04) ^a,b^ | < 0.001 |
| Word List Recognition, Z score | 0.57 (0.47 – 0.62) | 0.31 (0.0 – 0.69) | -1.87 (-2.6 – -0.62) ^a,b^ | -2.65 (-4.53 – -1.56) ^a,b^ | < 0.001 |
| Constructional Praxis Recall, Z score | 0.90 (0.033 – 1.04) | 0.21 (0.0 – 0.56) | -0.78 (-1.52 – -0.32) ^a,b^ | -1.92 (-2.08 – -1.32) ^a,b^ | < 0.001 |
| RCTF, 3 min delayed recall | 16.5 (13 – 20.5) | 12.5 (10.5 – 15.5) | 10 (6.5 – 16) ^a,b^ | 0 (0 – 2.5) ^a,b,c^ | < 0.001 |
| RCTF, 30 min delayed recall | 17 (15 – 20.5) | 12.5 (9.5 – 15.5) | 8.5 (4 – 12) ^a^ | 0 (0 – 0.5) ^a,b,c^ | < 0.001 |
| LM immediate recall | 23 (18 – 27) | 13 (10 – 15) ^a^ | 7.0 (5.0 – 13) ^a^ | 2 (1.0 – 5.0) ^a,b,c^ | < 0.001 |
| LM delayed recall | 6 (3 – 9) | 4.5 (3 – 7) | 2 (0 – 5) ^a^ | 1 (0 – 4) ^a^ | 0.0018 |
| LM recognition | 22 (21 – 25) | 18 (17 – 20) ^a^ | 17 (15 – 19) ^a^ | 14 (13 – 16) ^a,b^ | 0.0035 |
| Visuoconstruction, median (95% CI) | | | | | |
| Construction praxis, Z score | 0.43 (0.25 – 0.57) | 0.24 (-0.19 – 0.44) | 0.27 (-0.82 – 0.57) | -0.42 (-1.41 – 0.11) | 0.0797 |
| RCTF copy | 35 (33 – 35) | 33 (30 – 34) ^a^ | 32 (29 – 33) ^a^ | 18.5 (6 – 23.5) ^a,b,c^ | < 0.001 |
| Block design | 38 (33 – 45) | 28 (24 - 29) ^a^ | 28 (24 – 30) ^a^ | 11 (4 – 20) ^a,b,c^ | < 0.001 |
| Clock drawing | 30 (29 – 30) | 28.5 (27 – 29) ^a^ | 27 (26 – 29) ^a^ | 23 (16 – 26) ^a,b,c^ | < 0.001 |
| Executive function, median (95% CI) | | | | | |
| COWAT | 27 (23 – 30) | 20 (17 – 24) ^a^ | 18 (15 – 23) ^a^ | 8 (2 – 18) ^a,b^ | < 0.001 |
| FAB | 16 (15 – 17) | 15 (14 – 15) | 11 (10 – 13) ^a,b^ | 9 (5 – 12) ^a,b^ | < 0.001 |
| TMT-A (0 – 360 s) | 45 (37 – 52) | 56 (47 – 66) | 67 (50 – 83) ^a^ | 131.5 (69 – 218) ^a,b^ | < 0.001 |
| TMT-B (0 – 360 s) | 110 (85 – 138) | 173 (120 - 195) | 232 (151 - 300) ^a^ | 300 (113 - 360) ^a^ | < 0.001 |

^a^p<0.05 versus CN; ^b^p<0.05 versus SCD; ^c^p<0.05 versus MCI by Dunn’s multiple comparison following the Kruskal-Wallis test. LM, Logical Memory; RCTF, Rey Complex Figure Test; COWAT, Controlled Oral Word Association Test; FAB, Frontal Assessment Battery; TMT, Trail Making Test

**Table S2. Diagnostic Performance of CSF Biomarkers Measured by Three Platforms to Discriminate AD from CN.**

| **Assay Platforms** | **Parameters** | **Aβ_42_** | **T-tau** | **P-tau** | **T-tau/ Aβ_42_** | **P-tau/ Aβ_42_** | **Aβ_42_/ Aβ_40_** |
| --- | --- | --- | --- | --- | --- | --- | --- |
| Luminex-xMAP | ROC AUC | 0.907 | 0.763 | 0.816 | 0.913 | 0.897 | - |
|  | Cut-off value | 433.7 pg/mL | 57.77 pg/mL | 22.55 pg/mL | 0.136 | 0.050 | - |
|  | Sensitivity (%) | 91.3 | 69.6 | 78.3 | 91.3 | 82.6 | - |
|  | Specificity (%) | 79.3 | 82.8 | 93.1 | 100 | 100 | - |
| INNOTEST | ROC AUC | 0.876 | 0.870 | 0.798 | 0.927 | 0.912 | 0.922 |
|  | Cut-off value | 493.0 pg/mL | 240.0 pg/mL | 67.7 pg/mL | 0.565 | 0.081 | 0.090 |
|  | Sensitivity (%) | 87.0 | 86.4 | 57.1 | 86.4 | 90.5 | 91.3 |
|  | Specificity (%) | 89.7 | 75.0 | 92.3 | 100 | 92.3 | 86.2 |
| Lumipulse | ROC AUC | 0.889 | 0.943 | 0.927 | 0.952 | 0.946 | 0.952 |
|  | Cut-off value | 564.0 pg/mL | 320.5 pg/mL | 34.4 pg/mL | 0.453 | 0.053 | 0.059 |
|  | Sensitivity (%) | 76.2 | 85.7 | 95.2 | 85.7 | 90.5 | 90.5 |
|  | Specificity (%) | 96.4 | 92.9 | 78.6 | 100 | 100 | 96.4 |

**Table S3. CSF Biomarker Levels Measured by Different Immunoassay Platforms According to Amyloid PET Result.**

| **Assay Platforms** | **Parameters** | **Aβ_42_** | **Aβ_40_** | **T-tau** | **P-tau** | **T-tau/ Aβ_42_** | **P-tau/ Aβ_42_** | **Aβ_42_/ Aβ_40_** |
| --- | --- | --- | --- | --- | --- | --- | --- | --- |
| Luminex-AlzBio3 | Aβ PET – | 547.3  (520.5 – 563.5) | - | 47.2  (45.0 – 52.1) | 16.3  (15.1 – 18.0) | 0.09  (0.08 – 0.10) | 0.03  (0.027– 0.033) | - |
|  | Aβ PET + | 291.5  (243.1 – 378.1) | - | 64.1  (43.9 – 95.0) | 26.9  (19.0 – 38.8) | 0.22  (0.14 – 0.41) | 0.09  (0.06 – 0.17) | - |
|  | P value | < 0.001 | - | < 0.001 | < 0.001 | < 0.001 | < 0.001 | - |
| INNOTEST | Aβ PET – | 766.0  (706.6 – 841.2) | 7175  (6654 – 8064) | 191.7  (181.5 – 214.2) | 44.0  (39.2 – 46.7) | 0.26  (0.23 – 0.28) | 0.05  (0.05 – 0.06) | 0.11  (0.10 – 0.11) |
|  | Aβ PET + | 417.7  (340.5 – 474.2) | 7405  (5844 – 9108) | 375.9  (260.1 – 620.1) | 53.6  (46.5 – 85.2) | 1.04  (0.57 – 1.67) | 0.15  (0.10 – 0.25) | 0.06  (0.04 – 0.08) |
|  | P value | < 0.001 | 0.752 | < 0.001 | < 0.001 | < 0.001 | < 0.001 | < 0.001 |
| Lumipulse | Aβ PET – | 967.5  (898.6 – 1072) | 11885  (11081 – 12807) | 220.5  (193.0 – 236.0) | 28.1  (26.0 – 30.0) | 0.21  (0.19 – 0.23) | 0.03  (0.026– 0.030) | 0.09  (0.08 – 0.09) |
|  | Aβ PET + | 500.7  (441.8 – 606.2) | 11656  (9906 – 13419) | 379.0  (247.0 – 611.0) | 52.7  (37.2 – 103.5) | 0.95  (0.41 – 1.53) | 0.13  (0.07 – 0.24) | 0.05  (0.04 – 0.05) |
|  | P value | < 0.001 | 0.425 | < 0.001 | < 0.001 | < 0.001 | < 0.001 | < 0.001 |

**Table S4.** **Diagnostic Performance of CSF Biomarkers Measured by Luminex-Alzbio3 Platform to Discriminate Patients with AD from Healthy Controls (HC) Using Predetermined Cutoffs in an Independent Cohort.**

| Biomarker Level and Diagnostic Performance | | Aβ_42_ (pg/mL) | T-tau (pg/mL) | P-tau (pg/mL) | T-tau/ Aβ_42_ | P-tau/ Aβ_42_ |
| --- | --- | --- | --- | --- | --- | --- |
| Group | HC (n = 10) | 559.2  (471.5 – 663.5) | 59.5  (47.9 – 66.7) | 27.1  (18.3 – 42.5) | 0.102  (0.074 – 0.146) | 0.046  (0.029 – 0.084) |
|  | MCI (n = 7) | 380.0  (212.2 – 569.1) | 79.5  (50.3 – 104.9) | 44.5  (28.7 – 58.9) | 0.170  (0.021 – 0.561) | 0.090  (0.025 – 0.297) |
|  | AD (n = 17) | 232.0 ^a^  (195.1 – 337.9) | 85.5^a^  (69.9 – 108.7) | 31.2  (28.1 – 48.5) | 0.354 ^a^  (0.294 – 0.491) | 0.135 ^a^  (0.120 – 0.198) |
| P value* | | <0.001 | 0.0066 | 0.2430 | 0.0001 | 0.0016 |
| ROC AUC | | 0.941 | 0.877 | 0.629 | 0.977 | 0.912 |
| Sensitivity (%) | | 82.4 | 64.7 | 76.5 | 94.1 | 94.1 |
| Specificity (%) | | 90.0 | 90.0 | 40.0 | 90.0 | 80.0 |

* Kruskal-Wallis test followed by Dunn’s multiple comparison (^a^*P* < 0.05 vs. HC). Median [range] age of HC, MCI and AD were 71 [54 – 75], 75 [62 – 80] and 65 [53 – 75], respectively (p=0.015). Gender distribution (M:F) of HC, MCI and AD were 3:7, 4:3, and 11:6, respectively (p=0.212).
